# Charge shielding effects of PEG bound to NH_2_-terminated PAMAM dendrimers – an experimental approach†

**DOI:** 10.1039/d2sm01698b

**Published:** 2023-03-23

**Authors:** Brandon M. Johnston, Alan J. Grodzinsky, Paula T. Hammond

**Affiliations:** a Department of Chemical Engineering, Massachusetts Institute of Technology 77 Massachusetts Ave Cambridge MA 02139 USA hammond@mit.edu; b Koch Institute for Integrative Cancer Research 500 Main St Cambridge MA 02139 USA; c Department of Biological Engineering, Massachusetts Institute of Technology 77 Massachusetts Ave Cambridge MA 02139 USA; d Department of Electrical Engineering and Computer Science, Massachusetts Institute of Technology 77 Massachusetts Ave Cambridge MA 02139 USA; e Department of Mechanical Engineering, Massachusetts Institute of Technology 77 Massachusetts Ave Cambridge MA 02139 USA

## Abstract

Cationic poly(amido amine) (PAMAM) dendrimers exhibit great potential for use in drug delivery, but their high charge density leads to an inherent cytotoxicity. To increase biocompatibility, many studies have attached poly(ethylene glycol) (PEG) chains to the dendrimer surface. It is unclear how these tethered PEG chains influence the physicochemical properties of the dendrimer. Here, we develop a fluorescence-based assay utilizing anionic biological tissue to quantify the electrostatic binding affinity of a library of PEG–PAMAM conjugates with various PEG chain lengths and grafting densities. We find that covalently bound PEG chains reduce the electrostatic binding affinity more significantly than what can be achieved through covalent bonds only. Contrary to previous thought, this reduction is not explained by the steric hindrance effects of PEG chains, suggesting that other, non-covalent interactions between PEG and PAMAM are present. Using acetylated PAMAM conjugates, we convert electrostatic binding affinity to the number of charged amines accessible to the physiological environment. These data, coupled with ^1^H-NMR, allows us to study more closely the non-covalent interactions between PEG and PAMAM. We find that increasing PEG chain length increases the number of non-covalent interactions. Additionally, at low grafting densities, increasing the number of PEG chains on the PAMAM surface also increases the non-covalent interactions. At higher grafting densities, however, PEG chains sterically repel one another, forcing chains to elongate away from the surface and reducing the number of interactions between PAMAM and individual PEG chains. The data presented here provides a framework for a more precise mechanistic understanding of how the length and density of tethered PEG chains on PAMAM dendrimers influence drug delivery properties.

## Abstract 2

An experimental technique to probe intramolecular interactions of PEG–PAMAM conjugates and surface charge presentation. Increasing PEG chain length increases non-covalent shielding, but increasing chain density decreases PEG surface association.

## Graphical abstract

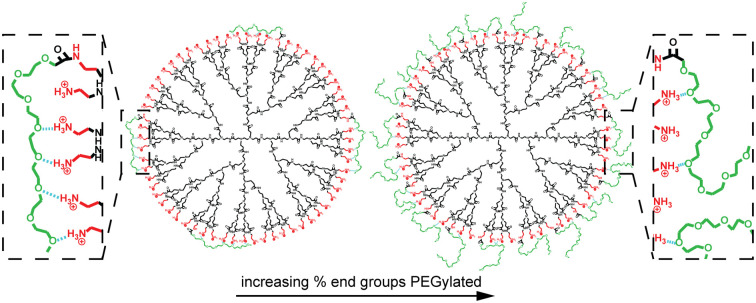

## Introduction

Poly(amido amine) (PAMAM) dendrimers are hierarchically branched macromolecules with a high density of peripheral primary amines. These commercially available polymers are synthesized using a series of repeating reactions, resulting in a very well defined, uniform structure, low polydispersity, and availability in a range of increasing sizes and number of surface primary amines.^1^ Additionally, under physiological conditions, the primary amines become protonated,^2^ giving them ideal properties for use in charge-enabled drug delivery applications.^3–7^

Due to their spherical shape and exponentially branched architecture, PAMAM dendrimers exhibit a dense distribution of primary amines over a relatively small hydrodynamic volume.^1^ As a result of the high charge density, these cationic dendrimers exhibit an inherent cytotoxicity in their unmodified form by disrupting cellular membranes through cationic toxicity.^8,9^ Many groups investigating the use of PAMAM dendrimers for biological applications have employed the use of poly(ethylene glycol) (PEG) functionalization to help mitigate concerns of cytotoxicity.^3,5,6,10–12^ In addition, PEGylation, or the covalent attachment of PEG chains onto the surface of a molecule or substrate, can help increase the efficacy and the circulation times of PAMAM dendrimers *in vivo*.^13,14^ Examples of PEG–PAMAM conjugates for biological applications include cancer therapeutics,^4,15,16^ gene delivery,^5–7^ imaging in diagnostic tests,^17^ and avascular tissue drug delivery.^3^

PEG–PAMAM conjugates are interesting drug delivery vehicles because of their topology, charge and engineered surface properties. These considerations affect how the dendrimer nanoparticle interacts with the physiological environment, ultimately enabling these systems to be tuned as delivery vehicles to cells and tissues with covalently bound drugs or moieties. For example, in our recent work, we found that PEG–PAMAM conjugates can be designed to electrostatically bind to anionic articular cartilage and increase the joint residence time of covalently bound proteins.^3^ Notably, the presence of unmodified cationic amines alone did not predict binding ability for these systems; depending on PEG chain length and percent conjugation, certain PEGylated conjugates did not interact with cartilage. This work inspired us to launch a thorough investigation into how PAMAM charge presentation can be manipulated with PEG to control transport within cartilage, including deep penetration and long-term residence. Experimental techniques such as small-angle neutron scattering and small-angle X-ray scattering can be employed to gain insight into the molecular structure of PEG–PAMAM conjugates,^18–21^ but these techniques fail to provide detail into important surface properties of these conjugates, such as how tethered PEG interacts with the dendrimer surface on a molecular level, the accessibility of primary amines, and ultimately the presentation of charge. These details are critical to more completely understanding PEG–PAMAM conjugates as a drug delivery platform and fine-tuning their surface properties for specific delivery applications, such as cartilage delivery.

To help gain a complete understanding of PEG–PAMAM conjugates, researchers have turned to computational methods.^22–25^ These computational studies have helped reconcile experimental data by identifying the open, segmented structure of PEG–PAMAM conjugates that result from a force balance between internal, dispersive interaction collapsing forces and external, electrostatic expansion forces.^24^ They have identified strong associations between covalently bound PEG chains and the PAMAM surface through hydrogen bonding of the PEG oxygen lone pair electrons (acceptor) and PAMAM primary amines (donor).^24,25^ These studies have helped to answer important questions about the molecular structure of PEG–PAMAM conjugates and the interactions between tethered PEG chains and the underlying PAMAM surface, but they fall short in understanding how the cationic PAMAM surface is presented to the environment.

PEG's ability to shield charge is a phenomenon that has been extensively used to increase biocompatibility and develop stealth coatings for nanoparticles.^13,26,27^ Here, we sought to understand how this shielding takes place in PEG–PAMAM conjugates using experimental techniques. We hypothesize PAMAM PEGylation will reduce the surface charge of the conjugate through both covalent and non-covalent shielding. There is some experimental evidence supporting this using zeta potential measurements,^10^ but this technique is insensitive to discrete changes in surface charge, such as those that arise from different dendrimer generations.^28,29^ Additionally, we hypothesize that longer PEG chains will reduce the surface charge more drastically than shorter PEG chains due to a greater number of non-covalent interactions between PEG and PAMAM. Some zeta potential measurements support this hypothesis as well,^10^ while others do not.^6^ Zeta potential falls short in reliable quantification of PEG–PAMAM surface charge due to the small size and ligand corona of these conjugates.^30,31^ There is a need for a quantitative and sensitive experimental technique to probe the nature of PAMAM charge shielding by PEG more accurately.

The objective of this work was to develop an experimental technique to quantify the reduction in accessible surface charge of PAMAM dendrimers covalently modified with PEG. First, a fluorescence-based assay utilizing a relevant dense anionic matrix, specifically articular cartilage, is investigated to quantify the relative electrostatic binding affinities of a library of PEG–PAMAM conjugates. A smaller library of acetylated PAMAM conjugates was used to convert relative binding affinities to number of charged amines on the surface of the dendrimer that are accessible to the physiological environment. An equation is then introduced describing the accessibility of PAMAM primary amines, which allows for quantification of the non-covalent interactions between PEG and PAMAM. Steric hindrance effects of PEG are discussed and modeled as an alternative hypothesis to non-covalent interactions being the driving force of PEG charge shielding effects. This alternate theory fails to reconcile with data, supporting our conclusions that non-covalent interactions are the driving force of PEG charge shielding effects. Using these data, we gain a greater understanding of the impact of PEG interactions with the PAMAM surface for a range of PEG chain lengths and PEG grafting densities.

## Materials and methods

### Materials

All reagents were used as received without further purification or modification unless otherwise specified. PAMAM dendrimers, generation four and generation six, with ethylenediamine cores, were obtained from Dendritech through Sigma as solutions in methanol (generation four – 10 wt% in methanol, generation 6–5 wt% in methanol). A generation ‘*X*’ PAMAM dendrimer, often denoted as GX PAMAM, has 2^*x*+2^ terminal, primary amines. For example, generation four PAMAM contains 64 primary amines, and generation six PAMAM contains 256 primary amines. Before use, PAMAM dendrimer solutions were transferred to a 100 mL round bottom flask for methanol removal *via* dry ice rotary evaporation. The dendrimer was then washed 4 times with 4 mL of DI H_2_O by ultracentrifugal filtration (Amicon Ultra 10k or 30k molecar weight cutoff, 4 mL, for G4 and G6, respectively). The pure dendrimer was then lyophilized and reconstituted in MilliQ H_2_O for future use. Methoxy PEG succinimidyl carboxymethyl ester (mPEG-SCM, *M*_W_ = 350 Da, 550 Da, 1 kDa, 2 kDa) were obtained from Creative PEGworks. Methoxy PEG succinimidyl propionate (m-PEG_13_ – NHS ester) was obtained from BroadPharm. Alexa Fluor™ 647 NHS ester (succinimidyl ester) was purchased from ThermoFisher Scientific. Deuterium oxide (99.9 atom% D, glass distilled), anhydrous methanol (99.8%), triethylamine (≥99.5%), and acetic anhydride (≥99.5%) were purchased from Sigma-Aldrich. Sodium bicarbonate (molecular biology grade), penicillin–streptomycin (100×), MEM non-essential amino acid solution (100×, without l-glutamine, cell culture grade), l-proline (non-animal source, cell culture grade), and ascorbic acid (20–200 mesh, cell culture grade) were purchased from Sigma. Dulbecco's Modified Eagle Medium (DMEM) without phenol red (4.5 g L^−1^ glucose, without sodium pyruvate), fetal bovine serum (qualified), (4-(2-hydroxyethyl)-1-piperazineethanesulfonic acid) (HEPES, 1 M), and sodium pyruvate (100 mM) were purchased from Gibco. Anhydrous dimethyl sulfoxide (DMSO, HPLC grade, 99.9+%) was purchased from Alfa Aesar. 1× phosphate-buffered saline (1× PBS, 154 mM NaCl, 5.6 mM Na_2_HPO_4_, 1.06 mM KH_2_PO_4_, ∼160 mM total salt, pH 7.4, without calcium or magnesium) was purchased from Lonza and 10× phosphate-buffered saline (10× PBS, 1368.9 mM NaCl, 26.83 mM KCl, 101.437 mM Na_2_HPO_4_, 17.64 mM KH_2_PO_4_, ∼1500 mM salt, pH 7.4, without calcium or magnesium, molecular biology grade) was purchased from Corning. All other PBS concentrations were generated by mixing 10× PBS with 1× PBS or 1× PBS with sterile water.

### Dendrimer-PEG reaction

PAMAM dendrimer solution (12.5 mM G4, 2.5 mM G6) was added to a sodium bicarbonate solution (1.0 M) to make a 10% v/v solution, the pH of which was then adjusted to approximately 8.0 with hydrochloric acid. A theoretical amount of PEG needed for the desired% end group PEGylation was calculated, assuming quantitative yields, and solubilized in anhydrous DMSO (100–200 μL). The dendrimer solution was then diluted with sodium bicarbonate solution (0.1 M) to ensure DMSO was not more than 10–20% of the final volume. The PEG/DMSO solution was added to the dendrimer solution, followed shortly by vortexing of the mixture. The reaction vessel was covered with foil and left for two hours at room temperature on a shaker plate. To purify the final product, the reaction mixture was washed 4 times with 4 mL of DI H_2_O by ultracentrifugal filtration (Amicon Ultra 10k or 30k molecular weight cutoff, 4 mL) in a swinging-bucket centrifuge at 3500 × *g*. The final product was collected and lyophilized to afford a white, fluffy solid for large amounts of PEG or clear, oily solid for small amounts of PEG. Products were reconstituted to 1000 μM in MilliQ H_2_O, and stored at 4 °C.

### Dendrimer acetylation reaction

An aliquot of PAMAM dendrimer solution (12.5 mM G4, 2.5 mM G6) was moved to a 4 mL scintillation vial and water was removed *via* dry ice rotary evaporation. The dendrimer was reconstituted in anhydrous methanol (3 mM) and the vial was charged with a stir bar. Vortexing and magnetic stirring was performed to create a homogeneous solution. A theoretical amount of acetic anhydride needed for the desired % end group acetylation was calculated, assuming quantitative yields, and added to the dendrimer in methanol solution while stirring, followed quickly by triethylamine (1.1 eq. of acetic anhydride). The solution was briefly vortexed and left to react at room temperature for 1 hour while stirring, capped. To purify the final product, the methanol was removed from the reaction vessel using dry ice rotary evaporation and the product was washed 3 times with 4 mL of 1× PBS and 2 times with 4 mL of DI H_2_O by ultracentrifugal filtration (Amicon Ultra 10k or 30k molecular weight cutoff, 4 mL) in a swinging bucket centrifuge at 3500 × *g*. The final product was collected and lyophilized to afford a clear, oily solid. Products were reconstituted to 1000 μM in MilliQ H_2_O, and stored at 4 °C.

### Fluorescent labeling of dendrimer conjugates

PAMAM conjugate solution (1.0 mM) was diluted in sodium bicarbonate solution (0.1 M) at a 1 : 2 v : v ratio, and vortexed. An aliquot of Alexa Fluor™ 647 NHS ester was thawed at room temperature, and reconstituted in anhydrous DMSO to a final solution concentration of 10 mg mL^−1^. Alexa Fluor™ 647 solution (10 mg mL^−1^) was added to the dendrimer solution (0.33 mM) to achieve a 1 : 1 molar ratio, and quickly triturated and vortexed and ensure homogenous mixing. The reaction vessel was covered with foil and left for two hours at room temperature on a shaker plate. To purify the final product, the reaction mixture was washed 4 times with 4 mL of 1× PBS by ultracentrifugal filtration (Amicon Ultra 10k or 30k molecular weight cutoff, 4 mL) in a swinging-bucket centrifuge at 3500 × *g*. The final product was collected and diluted in 1× PBS in a tared vessel. The final concentration of the fluorescently–tagged conjugate was calculated using the weight of the final solution, and stored at 4 °C while wrapped in foil. Fluorescent tag incorporation was quantified *via* Alexa Fluor™ 647 absorbance at 650 nm (*ε* = 270 000 M^−1^ cm^−1^) using the UV-vis function of a NanoDrop 1000 Spectrophotometer (ThermoFisher).

### Bovine cartilage explant harvest and culture

Cartilage was harvested from the trochlear groove of young (1–2 weeks old) bovine knee joints obtained from Research 87 (Hopkinton, MA) within 12 hours of euthanasia. Explant biopsies with a 3 mm diameter were removed from intact cartilage, washed with PBS, and trimmed into uniform cylinders (height = 1 mm, diameter = 3 mm) containing intact superficial zone tissue (∼200 μm) and middle zone cartilage (∼800 μm).^32–34^ Explants were immediately transferred to 96 multi-well plates containing DMEM (without phenol red), 10% v/v fetal bovine serum (FBS), 1% v/v penicillin–streptomycin (PS), 1% HEPES buffer, 1% non-essential amino acids, 1% sodium pyruvate, and supplemented with 0.4% fresh l-proline and 0.4% ascorbic acid. Explants were cultured at 37 °C and 5% CO_2_ conditions for at least 24 hours prior to any experiments to allow tissue recovery from the harvesting procedure. The media was changed every other day, and no explants used in the study were cultured for more than two weeks to ensure only healthy tissue was used.

### Proton nuclear magnetic resonance (^1^H NMR)

PEGylation of PAMAM dendrimers was characterized using ^1^H NMR (Bruker AVANCE, 400 MHz or 500 MHz). H_2_O was removed from samples *via* dry ice rotary evaporation and reconstituted in D_2_O. Proton chemical shifts are reported in ppm (*δ*). The PEG grafting density was determined from the integral ratio between PEG ethylene (OC**H**_**2**_C**H**_**2**_) and PAMAM methylene (NCH_2_C**H**_**2**_CONH) protons. ^1^H NMR (D_2_O) *δ* 2.39–2.44 (PAMAM, NCH_2_C**H**_**2**_CONH), *δ* 2.61–2.68 (PAMAM, CH_2_C**H**_**2**_N(CH_2_)_2_), *δ* 2.80–2.84 (PAMAM, NC**H**_**2**_CH_2_CONH), *δ* 3.00–3.15 (PAMAM, NH_2_C**H**_**2**_C**H**_**2**_NH), *δ* 3.36–3.39 (PEG, OC**H**_**3**_), *δ* 3.68–3.71 (PEG, OC**H**_**2**_C**H**_**2**_), *δ* 4.05–4.08 (PAMAM–PEG, NHCOC**H**_**2**_O). Acetylation of PAMAM dendrimer was characterized using ^1^H NMR (Bruker AVANCE, 400 MHz or 500 MHz). H_2_O was removed from samples *via* dry ice rotary evaporation and reconstituted in D_2_O. Proton chemical shifts are reported in ppm (*δ*). The percent of dendrimer end groups acetylated, or degree of acetylation, was determined from the integral ratio between acetyl methyl (NHCOC**H**_**3**_) protons and PAMAM methylene (NCH_2_C**H**_**2**_CONH) protons. ^1^H NMR (D_2_O) *δ* 1.93–2.03 (acetyl, NHCOC**H**_**3**_), *δ* 2.39–2.44 (PAMAM, NCH_2_C**H**_**2**_CONH), *δ* 2.61–2.68 (PAMAM, CH_2_C**H**_**2**_N(CH_2_)_2_), *δ* 2.80–2.84 (PAMAM, NC**H**_**2**_CH_2_CONH), *δ* 3.1–3.55 (PAMAM, NH_2_C**H**_**2**_C**H**_**2**_NH). The accessible charged amines of acetylated formulations were calculated by subtracting the number of acetyl groups covalently bound to the surface from the total PAMAM end groups available:

1 amines_accessible,acetylation_ = end groups_total_ − number of end groups acetylated

### Quantification of conjugate accessible charged amines

Ten bovine cartilage explants were incubated with fluorescently-labeled PAMAM conjugates (200 μL, 1.25 uM) in DMEM (without phenol red), 10% FBS, 1% PS, 1% non-essential amino acids, 1% sodium pyruvate, and 1% HEPES buffer supplemented with 0.4% fresh l-proline and 0.4% ascorbic acid in a black bottom, black chimney Nunc™ F96 MicroWell™ polystyrene plate (ThermoFisher). In addition, two wells of fluorescently-labeled dendrimer were added without cartilage. Plate 1 incubation was performed for 24 hours at 37 °C and 5% CO_2_. After incubation, each of the ten cartilage explants were removed from the conjugate baths and transferred to plate 2 containing warm salt baths (200 μL) of ten different PBS concentrations ranging from 0.5× PBS (75 mM salt) to 10× PBS (1500 mM salt). The fluorescence remaining in plate 1 was read using a Tecan Infinite 200Pro plate reader after the initial 24 hour incubation. Incubation of plate 2 was also performed for seven days at 37 °C and 5% CO_2_ with salt solutions being replaced with fresh solution after every 24 hours. The fluorescence remaining in plate 2 was measured after the first 24 hour incubation. The concentration of the remaining dendrimer in each well plate was determined according to calibration standards. The concentration of dendrimer remaining in plate 1 was used to calculate the percent uptake of the dendrimer conjugates according to the following equation:2
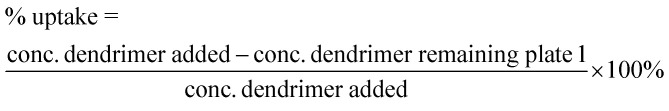


The concentration of dendrimer added was determined *via* the fluorescent wells without cartilage explants. The concentration of dendrimer remaining in plate 2 was used to calculate the percent desorption of the dendrimer conjugates at various salt concentrations according to the following equation:3
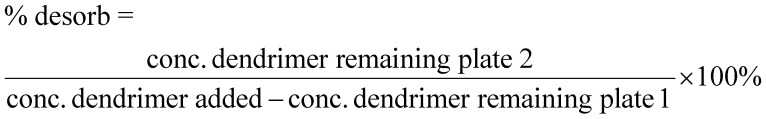


The critical salt concentration, defined as the minimum salt concentration necessary to screen enough electrostatic interactions between dendrimer conjugates and cartilage to begin dendrimer desorption, was determined by plotting percent desorption of the ten explants *versus* salt concentration. The salt concentration at which percent desorption begins to increase is the critical salt concentration. The critical salt concentration for PAMAM, both generation four and six, with a range of degrees of acetylation was determined and plotted against accessible charged amines as determine *via*^1^H-NMR. The resulting linear equation serves as a calibration curve to convert PEGylated PAMAM critical salt concentration to accessible charged amines.

## Results and discussion

In this study, we sought to use quantitative and sensitive experimental techniques to understand how PEG chains covalently bound to the surface of PAMAM dendrimers influence the downstream physicochemical properties based on charge. While it has been hypothesized, and computationally determined, that PEG chains are able to non-covalently interact with and shield the underlying PAMAM surface,^24,25^ this concept has not been experimentally confirmed. In particular, the lack of direct observation of shielding effects is largely due to limitations associated with reliable zeta potential measurements of PEG–PAMAM conjugates, due to their small (<10 nm) size approaching the limit of detection from Brownian motion effects and the presence of a dynamic ligand corona whose conformation greatly impacts surface charge shielding.^30,31^ Here, generation four and six cationic PAMAM dendrimers have been functionalized with mPEG or acetyl groups with various degrees of surface functionalization and PEG chain lengths. These conjugates have been characterized *via* proton NMR and a fluorescence-based assay probing the strength of electrostatic interactions with a highly consistent and biologically relevant anionic matrix, bovine cartilage explants. Using the results obtained from acetylated dendrimers – which are unable to form non-covalent interactions with free amines on the PAMAM surface – as a baseline, we can experimentally quantify the number of accessible charged amines on the surface of the conjugate. Ultimately, this study quantifies the non-covalent interactions between PEG and PAMAM for a library of conjugates with varying PEG chain lengths and PEG grafting densities. These results give us insight into how tethered PEG chains alter the presentation of the underlying PAMAM surface to the physiological environment.

### Using *ex vivo* cartilage and salt to probe electrostatic binding affinity of PEGylated dendrimers

**Fig. 1:**
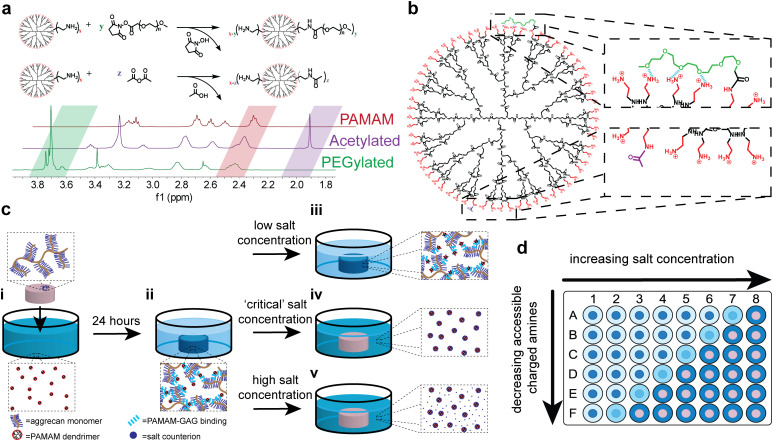
Theoretical basis and experimental schematic behind using salt-screening and acetylation to probe accessible charged amines of PEGylated dendrimers. (a) Synthetic schemes for the synthesis of PEGylated PAMAM formulations and acetylated PAMAM formulations with representative ^1^H-NMR spectra. (b) Schematic displaying how PEG chains (green) shield PAMAM amines (red) through non-covalent (blue) and covalent interactions but acetyl groups (purple) screen PAMAM amines through covalent interactions only. (c), (i) Well-defined, 3 mm diameter by 1 mm thick cartilage explants are submerged in a bath of fluorescent dendrimer. (ii) After 24 hours, the cationic, fluorescent dendrimers absorbs to the anionic aggrecan chains. (iii) At low salt concentrations, some dendrimer–aggrecan interactions are screened, but the dendrimer will remain adsorbed. (iv) At a critical salt concentration, enough dendrimer accessible charged amines are screened (blue circles complexed with red dendrimers) so that the dendrimer will begin to desorb from cartilage. (v) At high salt concentrations, all dendrimer accessible charged amines are screened with extra salt counter-ions (blue circles) in the solution. (d) Dendrimer absorption and release can be monitored using fluorescence in a high-throughput manner.

PAMAM dendrimer PEGylation can be readily achieved using NHS ester–amine chemistry, with the methoxy-capped PEG grafting density easily tuned *via* reaction stoichiometry. Similarly, dendrimer acetylation can be achieved through reaction with acetic anhydride, with the degree of acetylation easily modified by controlling the stoichiometry. These reactions, which can be characterized *via*^1^H-NMR to quantify the degree of end group modification, are compared in Fig. 1(a). The characteristic PAMAM methylene peak at 2.4–2.5 ppm can be compared to either the characteristic ethylene peak of PEG repeat units or methyl group peak of the acetyl groups, at 3.7–3.8 ppm or 2.0 ppm, respectively. Here, the shorthand abbreviation for PEGylated formulations is G*X̲*P*Y̲-Z̲*%, where ‘*X*’ is the dendrimer generation, ‘*Y*’ is the PEG chain length given as degree of polymerization, and ‘*Z*’ is the PEG grafting density. Acetylated formulations are denoted as *A*% acetylated, where ‘*A*’ is the degree of acetylation. A key difference between PEGylated and acetylated formulations, shown in Fig. 1(b), is that covalently bound PEG chains are hypothesized to interact with neighboring primary amines through hydrogen bonding, whereas covalently bound methyl groups do not. This distinction is critical to using acetylated formulations as a control to learn more about PEGylated formulations.

To quantify relative electrostatic binding affinity of PAMAM conjugates, fluorescently–labeled conjugates were left to interact electrostatically with the anionic aggrecan chains of articular cartilage explants in a high-throughput manner using a 96-well plate (Fig. 1(d)). All experiments were performed under physiological conditions at pH 7.4 and 37 °C. Under these conditions, both anionic aggrecan chains and cationic primary amines of the dendrimer are fully charged.^2,35^ After 24 hours, the amount of dendrimer removed from solution by the anionic matrix was determined *via* fluorescence quantification (Fig. 1(c)-ii). Following the 24 hours period of dendrimer uptake by the matrix, salt solutions with varying concentrations were introduced to the dendrimer–cartilage system for another 24 hours. The amount of dendrimer released from the anionic matrix into the salt solution after 24 hours was determined *via* fluorescence quantification and compared to the amount initially removed from the uptake solution to determine the percent of dendrimer desorbed from cartilage at various salt concentrations. At very low salt concentrations, the electrostatic interactions present between dendrimer and cartilage are not disrupted, and the dendrimer remains bound to the cartilage (Fig. 1(c)-iii). If electrostatic interactions are not present, the dendrimer is removed from the cartilage upon dilution by the low salt solution, in accordance with Donnan theory.^36,37^ Conversely, at sufficiently high salt concentrations, electrostatic interactions between dendrimer and cartilage would be screened entirely by salt counter-ions, leading to the desorption of dendrimer from the cartilage explants (Fig. 1(c)-v). Thus, for each modified dendrimer, at a conjugate-specific salt concentration enough electrostatic interactions between conjugate and dendrimer will be screened to begin dendrimer desorption (Fig. 1(c)-iv). This salt concentration is defined as the critical salt concentration of that specific dendrimer conjugate. Salt solutions were replaced with fresh solution every 24 hours for seven days to ensure the trends found in the critical salt concentration as a function of PAMAM functionalization were a result of thermodynamic effects only and that kinetic effects of desorption can be ignored. At the physiological salt concentration, daily replenishment of the salt bath resulted in minimal desorption of dendrimer from cartilage until a high salt concentration (ten times physiological levels) was introduced to screen the electrostatic interactions (Fig. S3, ESI†). For dendrimer conjugates with both high and low critical salt concentrations, replacing the salt baths daily had no effects on the critical salt concentrations (Fig. S4, ESI†) proving that kinetic effects of desorption can be ignored and that the cartilage explants remain intact at high salt concentrations over the seven day period.

The critical salt concentration gives information about the electrostatic binding affinity of a PEG–PAMAM conjugate to cartilage. If a conjugate has a weak electrostatic binding affinity, few salt counter-ions are required to disrupt binding, leading to a lower critical salt concentration. If strong electrostatic binding is present, more salt counter-ions are required, leading to a higher critical salt concentration. In this way, probing critical salt concentration for a library of PEG–PAMAM conjugates will expose differences in surface characteristics of the different formulations.

**Fig. 2:**
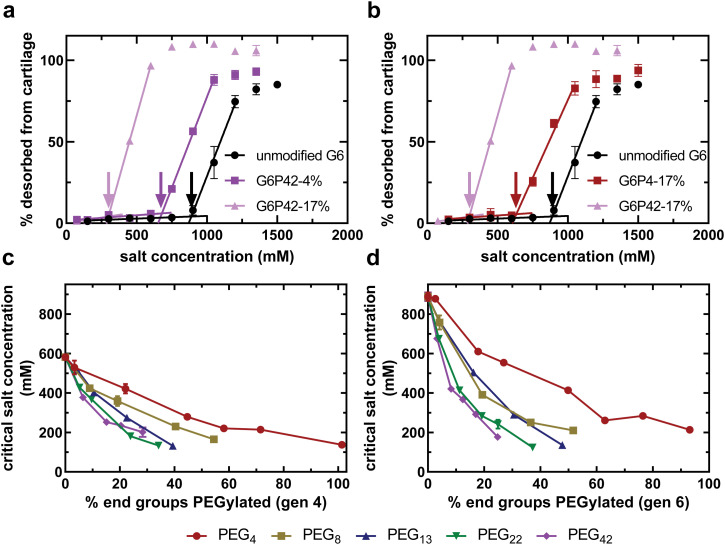
Increasing PEG grafting density or chain length results in a decrease in the critical salt concentration. (a) Differences in critical salt concentration with increasing PEG grafting density. (b) Differences in critical salt concentration with increasing PEG chain length. Arrows indicate the critical salt concentration of the corresponding PAMAM formulation. Critical salt concentration data for a library of PEGylated generation 4 PAMAM (c) and generation 6 PAMAM (d) as a function of % end groups PEGylated with varying PEG chain lengths. All data are means and standard deviation of 2 biological replicates with *n* = 1 technical replicate per animal.

Fig. 2(a) and (b) compare the dendrimer desorption results for PEG–PAMAM conjugates with various PEG chain lengths and PEG grafting densities. As can be seen for all experiments, at lower salt concentrations, there is no desorption of the dendrimer conjugate from the cartilage explant. As the salt concentration is increased, a critical value is reached, defined here as the critical salt concentration, where the conjugate begins to desorb from cartilage. This critical salt concentration is determined by the onset of increased desorption of dendrimer from cartilage, indicated by the color-coded arrows in Fig. 2(a) and (b). At high salt concentrations, all conjugates have completely desorbed from cartilage. Similar curves are available for all experimental formulations in the ESI.†

As expected, incorporating PEG chains onto the surface of the dendrimer reduced the critical salt concentration, regardless of the chain length or density (Fig. 2). PEG chains are covalently bound to the dendrimer using an NHS ester tag that reacts with primary amines of the dendrimer to form an uncharged amide bond. This uncharged amide bond therefore eliminates a single primary amine from becoming protonated and participating in electrostatic interactions with cartilage, thus reducing the electrostatic binding affinity, and ultimately the critical salt concentration; however, there is also an additional effect that is evident as a function of the length of the PEG chain. Increasing the number of PEG chains of any length reduces the critical salt concentration further (Fig. 2(a)). Furthermore, keeping the number of PEG chains constant and increasing the PEG chain length also reduces the critical salt concentration (Fig. 2(b)). This finding suggests that there are non-covalent interactions between PEG and PAMAM that are shielding cationic amines from interacting with the anionic matrix. These non-covalent interactions increase with PEG chain length.

For a library of generation four (Fig. 2(c)) and generation six (Fig. 2(d)) PAMAM conjugates, as the number of PEG groups covalently bound to the surface of the dendrimer increases, the critical salt concentration decreases for all PEG chain lengths. The concentration of negatively-charged groups in articular cartilage is on the order of 100 mM^38^ while the concentration of positively-charged groups in the most cationic dendrimer conjugate solution tested in this study is 320 μM. Therefore, any difference in critical salt concentration is due to differences in the number of cationic charges accessible to the physiological environment, not differences in the anionic charges available in cartilage. This conjugate property is described as the accessible charged amines.

We tested PEG chains with lengths varying from a short PEG oligomer with only four repeat units to a longer PEG chain with 40 repeat units. Longer PEG chains result in a lower critical salt concentration (Fig. 2(b)–(d)). Since critical salt concentration is dependent on accessible charged amines, the longer PEG chains create a reduction in accessible charged amines that exceeds the expected observation from removing charged amines exclusively through covalent modification. This finding proves that PEG chains are capable of forming non-covalent interactions with surrounding primary amines of the PAMAM surface, shielding them from interacting with the negative charges of articular cartilage and, ultimately, the physiological environment.

The preceding analysis relies on the assumption that PEG chain length and grafting density impact electrostatic binding affinity solely by influencing the quantity of accessible charged amines. Electrostatic binding to articular cartilage depends on the chemical structure of the anionic binding site and cationic nanocarrier.^36^ For example, a study found that insulin-like growth factor 1 modified with a heparin-binding domain binds better to heparin sulfate anionic sites over chondroitin sulfate anionic sites.^39^ Here, it is assumed that PEG is completely inert to electrostatic binding and that all accessible charged amines of all conjugates investigated in this study possess equal ability to bind to the anions of cartilage.

### Using acetylation to quantify accessible charged amines

PEG chains bound to the surface of the dendrimer will interact with the primary amine end groups of the PAMAM surface through covalent and non-covalent interactions. The primary amines not interacting with the PEG chains are accessible to the physiological environment. Thus, for a PEGylated formulation, the PAMAM end groups can be described by the following equation:

4 end groups_total_ = amines_accessible_ + covalent bonds + non-covalent bonds

The covalent bonds between PEG and PAMAM can be calculated using proton NMR. Assuming defect-free PAMAM structures, the total end groups for generation four and six is 64 and 256, respectively. The result is one equation with two unknowns: the number of accessible charged amines and non-covalent interactions between PEG and PAMAM. To achieve a complete understanding of how PEG is interacting with and influencing the underlying PAMAM surface, which will impact the viability of the system for drug delivery applications, the number of accessible charged amines must be known.

Data obtained from the fluorescence assay developed for this study suggest that the critical salt concentration is dependent on the number of accessible charged amines on the surface of the dendrimer, but it is not possible to quantify directly the number of amines engaging in electrostatic interactions. Instead, an indirect method was devised using acetylated PAMAM conjugates. Through acetylation, primary amines are converted to amide bonds, leaving a methyl group on the surface of the dendrimer. With the assumption that short acetyl groups are incapable of non-covalently interacting with surrounding primary amines, the PAMAM end groups of the acetylated formulations can be described by the following equation:

5 end groups_total_ = amines_accessible_ + covalent bonds

Similar to PEGylation, proton NMR can be used to calculate the covalent bonds between acetyl groups and PAMAM. The result is one equation with one unknown: the accessible charged amines of the acetylated PAMAM conjugates. Therefore, the accessible charged amines of the acetylated formulations can be directly calculated using proton NMR data.

An assumption must be made that PEG chains and acetyl groups covalently bound to the surface of the dendrimer do not influence the free energy of electrostatic interactions. This means any changes in free energy of different conjugates interacting with cartilage is solely due to differences in the number of accessible charged amines. With this assumption, the relationship between accessible charged amines and critical salt concentration of the acetylated formulations can be used as a standard curve to convert critical salt concentration of the PEGylated formulations to accessible charged amines.

**Fig. 3:**
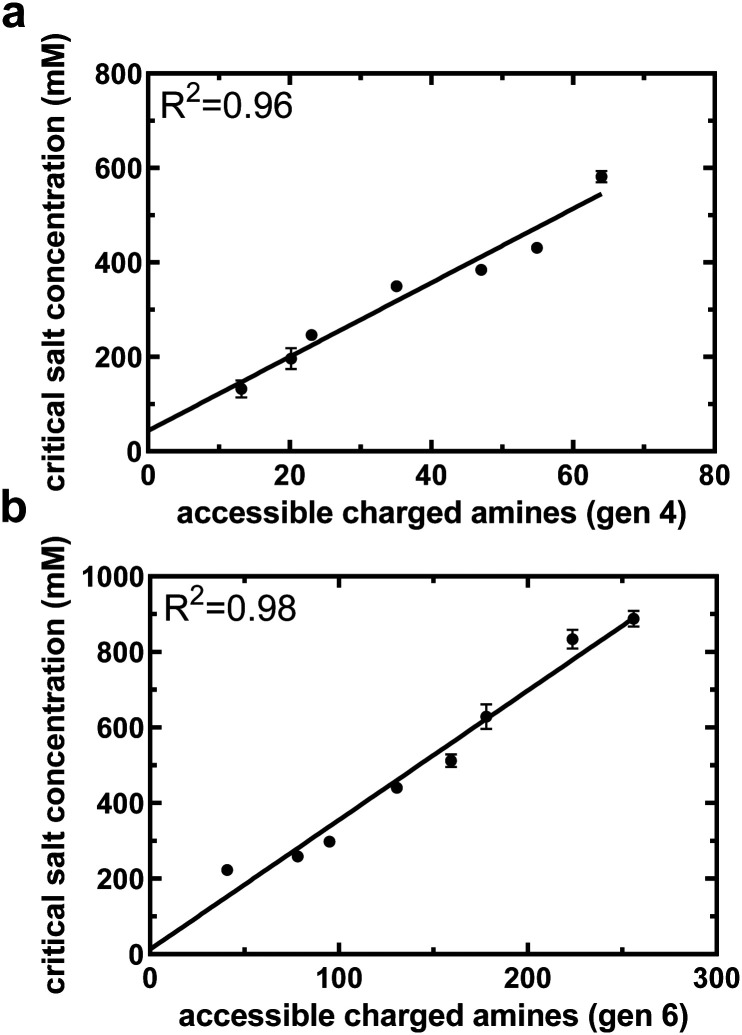
Critical salt concentration data of acetylated PAMAM can be used to help quantify accessible charged amines from critical salt concentration. Critical salt concentration for both generation 4 (a) and generation 6 (b) acetylated PAMAM dendrimers as a function of accessible charged amines, as determined by NMR and eqn (5). The linear relationship will aid in translating critical salt concentration to accessible charged amines for PEGylated formulations. All data are means and standard deviations of 2 biological replicates with *n* = 1 technical replicate per animal.

A small library of acetylated formulations was synthesized for this purpose, ranging from 0 to 84% of end groups acetylated. Fig. 3(a) and (b) displays the relationship between critical salt concentration and accessible charged amines for varying degrees of acetylation of PAMAM generation four and six, respectively. For both generations, the relationship between the critical salt concentration and the accessible charged amines, obtained from NMR data and calculated based on degree of acetylation, is linear (*R*^2^ = 0.96 and 0.98 for G4 and G6, respectively) with a *y*-intercept near zero. This validates our assumption that acetyl groups do not form non-covalent interactions with the underlying PAMAM surface and this linear relationship for the acetylated formulations can be used as a standard curve to convert critical salt concentration of the PEGylated formulations to accessible charged amines. Eqn (4), describing the accessibility of primary amines for PEGylated PAMAM formulations, now has a single unknown: the number of non-covalent interactions between PEG and PAMAM.

Using ionic matrices and salt concentration gradients is standard practice for ion exchange chromatography,^40^ commonly used for purification and recently employed to characterize the shielding effects of PEG in a mono-PEGylated peptide system.^41^ Utilizing *ex vivo* explants of anionic extracellular matrices and salt concentration gradients for the characterization of multivalent cationic nanoparticles is unique. Articular cartilage has, however, been described using ion exchange theory.^42^ Benefits of such an *ex vivo* characterization technique include: the ability to probe relevant biologic interactions, the reproducible and homogenous structure of the matrix, and the ease of acquiring the anionic matrix with few specialized tools and without chemical synthesis. We hypothesize the developed protocol for accessible charged amines characterization can be adapted to any anionic matrix and cationic molecule as long as the following conditions are met: (1) the concentration of anions is far greater than the concentration of cations and (2) a control cationic carrier can be obtained with a known number of accessible charged amines. This would allow the protocol to be used for a number of other systems, including the use of standard ion exchange chromatography equipment with sufficiently high ion concentrations.

### Reduced accessible charged amines through non-covalent interactions

**Fig. 4:**
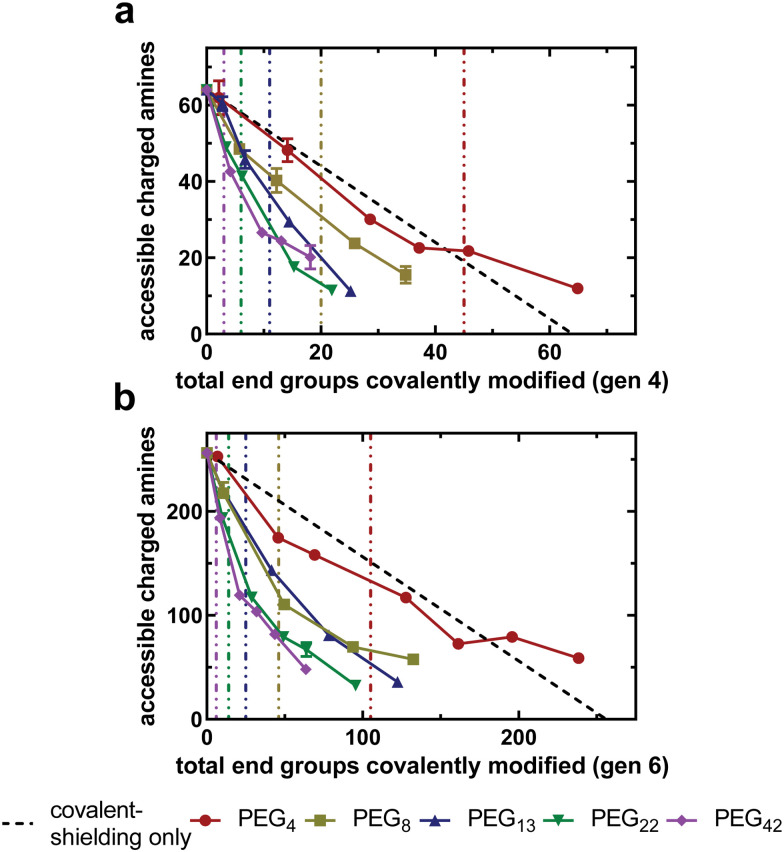
PEG chains shield amines through covalent and non-covalent interactions with the underlying PAMAM surface. Accessible charged amines on the underlying PAMAM surface as a function of the total PEG chains covalently bound to the surface for generation 4 (a) and generation 6 (b) PAMAM dendrimers. Vertical lines represent the PEG overlap number for the color corresponding PEG chain length. Each point represents the mean and standard deviation of 2 biologic replicates with *n* = 1 technical replicate per animal.

With an experimental protocol available to determine the number of non-covalent interactions between PEG and PAMAM, we can investigate how PEG chains of varying lengths interact with the underlying PAMAM surface at different grafting densities. Fig. 4(a) and (b) compares the relationship between the accessible charged amines of acetylated and PEGylated PAMAM conjugates as a function of the total amines covalently modified for the generation four and six libraries, respectively. The covalent-shielding only curve, represented by the black line and obtained using acetylated PAMAM, is the relationship between accessible charged amines and covalently bound acetyl groups. Any deviation from this covalent-shielding only curve is due to a change in the non-covalent interactions between PEG and PAMAM.

For all PEG chain lengths tested, there is a monotonic decrease in accessible charged amines as the PEG density increases. For all generation-chain length combinations tested, reduction in accessible charged amines was most drastic at low grafting densities (approaching zero), evident by the most negative slope at low grafting densities in Fig. 4(a) and (b). This trend is more prominent in generation six dendrimers (Fig. 4(b)), possibly because of the greater number of available primary amine end groups of the unmodified dendrimers. The slope of the curves begins to approach zero as the number of covalently modified end groups increases. This suggests that PEG chains at a lower grafting density are capable of forming a greater number of non-covalent interactions with PAMAM than at higher grafting densities, likely due to PEG inter-chain steric repulsion at higher densities. Longer PEG chains are able to reduce the number of accessible charged amines more dramatically than shorter PEG chains at all grafting densities, as there are more oxygen-containing PEG repeat units present to form hydrogen bonds with PAMAM primary amines.

The data presented here support the hypothesis that PEG chains are able to shield the PAMAM surface *via* non-covalent interactions, likely hydrogen bonding between PEG oxygen and PAMAM primary amines. This results in a reduced electrostatic binding affinity beyond what is achieved through covalent bonds only. An alternative hypothesis that would exhibit similar trends is that the PEG chains, tethered to the surface of the dendrimer, are sterically shielding the anionic charges of the extracellular matrix from electrostatically interacting with the underlying PAMAM cationic amines. The steric hindrance would increase, and electrostatic binding affinity decrease, as the PEG chain length and grafting density increases.

According to the work of Alexander,^43^ de Gennes,^44,45^ and others,^46,47^ three PEG chain structural regimes would be present in this steric hindrance PEG shielding model, defined by the morphology of the surface bound PEG chains as it relates to a critical overlap grafting density. The critical overlap grafting density is defined as the density at which the root mean squared end-to-end distance of neighboring grafted PEG chains begin to overlap. At low grafting densities, specifically below the critical overlap density, some dendrimer primary amines are permanently unshielded and able to interact freely with solution. This leads to a constant electrostatic potential in those areas and subsequent binding of dendrimer to the anionic matrix. At the critical overlap grafting density, PEG chains interact at the edge of their statistical radii, implying that all charged amines are shielded. However, as PEG chains are subject to kinetic fluctuations, the charged amines would be transiently exposed beneath the PEG chains and allow for limited electrostatic interactions with the anionic matrix. At grafting densities beyond the critical overlap density, PEG chains become confined to a volume of shorter width than their statistical diameter due to inter-chain steric repulsion, causing them to elongate away from the dendrimer. The result is a PEG “brush” that forms from a thermodynamic balance between entropic spring forces and steric repulsion from neighboring chains. This brush sterically inhibits the anionic matrix from interacting with the underlying PAMAM surface. As the grafting density increases further, inter-chain repulsion dominates, resulting in a stiff brush and more effective steric repulsion of the anionic matrix. Therefore, this model indicates that electrostatic interactions between PAMAM and the anionic matrix should be inhibited by PEG as the grafting density moves beyond the critical overlap grafting density.

In order to apply this model to the PAMAM–PEG system, the theory requires a number of assumptions and approximations. PAMAM must be approximated as a solid sphere with radius equal to the radius of gyration as determined by small-angle neutron scattering experiments.^48^ Because of this approximation, it is assumed that the primary amine end groups of PAMAM exist only on the surface of the sphere and are equidistant and stationary with respect to one another. It is also assumed that PEG chains are unable to exist within the sphere. Lastly, as an alternative hypothesis to the existence of non-covalent interactions, it is assumed that PEG chains do not interact with the surface of the sphere. Once these assumptions are made, the critical overlap grafting density of all generation-chain length combinations is calculated using the following equation:^43,44^6
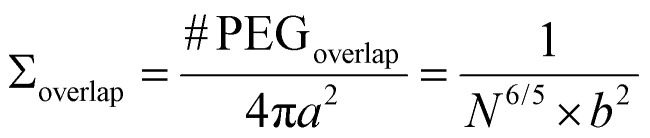
where *Σ*_overlap_ is the critical overlap grafting density (defined as number of grafted PEG chains per spherical dendrimer surface area), *a* is the radius of the dendrimer (determined by SANS^48^), *N* is the number of statistical Kuhn segments in the PEG polymer, and *b* is the statistical Kuhn length of PEG. The scaling of *N* assumes the aqueous environment is a good solvent for PEG chains. Details of these values and calculations can be found in Table S1 (ESI†). Also represented in Fig. 4(a) and (b) as vertical, color-coded lines are the calculated critical overlap PEG grafting densities for all ten generation-chain length combinations, as determined by the Alexander-de Gennes theory of polymer brushes discussed here.

Shorter PEG chains, such as PEG_4_ and PEG_8_, exhibit a low number of accessible charged amines at the critical overlap grafting density. Longer chains, however, such as PEG_22_ and PEG_42_, exhibit a high number of accessible charged amines at the critical overlap grafting density. For these longer chains, the grafting density must be four or five times the critical overlap grafting density to achieve relatively low accessible charged amines, indicating that PEG chains are not freely fluctuating around their statistical radii. The result is that the grafting density needed to shield the cationic surface completely is increased relative to what is predicted by the theory. This finding disproves the alternative hypothesis that steric shielding of the underlying PAMAM surface by PEG chains is the driving force behind the reduction in electrostatic binding affinity observed by increasing PEG chain length or grafting density.

### Effect of PEG chain length on non-covalent interactions

**Fig. 5:**
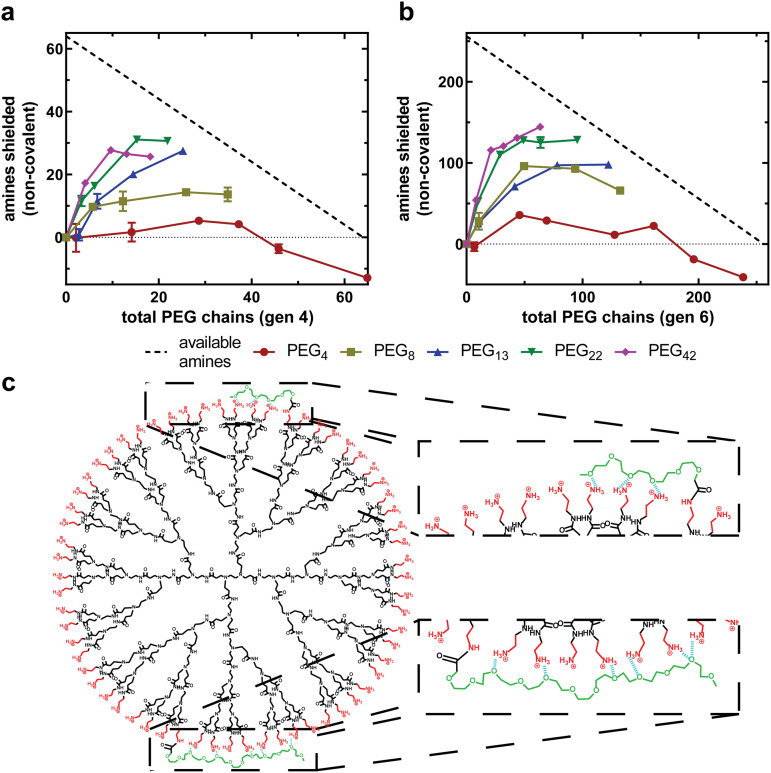
Increasing PEG chain length increases number of amines non-covalently shielded. Amines shielded through non-covalent interactions between PEG and PAMAM as a function of total PEG chains for generation 4 PAMAM (a) and generation 6 PAMAM (b). The negative values at high PEG 4 density are likely due to limitations associated with PEGylation quantification while assuming defect free dendrimers and monodisperse PEG chains, which is exacerbated as accessible charged amines and non-covalent interactions both approach zero. The number of PEG chains (*x*-axis) is also equivalent to the number of PAMAM amines shielded through covalent bonds between PAMAM and PEG. The black, dotted line represents the maximum number of non-covalently shielded amines possible for a given number of PEG chains (*X* – PEG chains = maximum, where *X* is the total number of primary amines for a given generation). All data are means and standard deviation of 2 biological replicates with *n* = 1 technical replicate per animal. (c) Visual representation of how longer PEG chains are capable of forming more non-covalent interactions with PAMAM amines than shorter PEG chains.

The accessible charged amines for each PEGylated formulation can now be input into eqn (4) to quantify the non-covalent interactions between PEG and PAMAM. Fig. 5(a) and (b) compares the number of non-covalent interactions for dendrimer conjugates with varying PEG chain lengths as a function of total PEG chains for generation four and generation six, respectively. As observed previously, for all generation-chain length combinations, the increase in non-covalent interactions is most substantial at low PEG grafting densities, followed by a plateau and, for some combinations, a decrease in non-covalent interactions with increasing PEG chains. This indicates that PEG chains are capable of interacting more strongly with the PAMAM surface at low PEG grafting densities than at high grafting densities (Fig. 5(c)), forming a PEG corona that closely associates with the PAMAM surface. The negative values at very high PEG 4 densities are likely due to limitations associated with PEG density quantification while assuming defect free dendrimers and monodisperse PEG, which would have the greatest effect at very high grafting densities and short PEG chain lengths when the accessible charged amines and non-covalent interactions both approach zero.

Additionally, longer PEG chains exhibit more non-covalent interactions with PAMAM than shorter PEG chains. This is observed as a more substantial deviation from the covalent-only curve in Fig. 4(a) and (b) but is quantified in Fig. 5(a) and (b). When PEG 4 is conjugated onto both generations tested, the chains interact very weakly with PAMAM, with at most 15% of the primary amines of PAMAM being shielded by PEG chains (G6P4-20%). PEG 42, however, is capable of non-covalently shielding over 50% of the primary amines (G4P42-10%). This is to be expected, as longer PEG chains have a greater number of repeat units, each with a single oxygen capable of forming hydrogen bonds with PAMAM primary amines. This also suggests that PEG repeat units are able to associate closely with PAMAM, regardless of their distance from the covalent attachment.

### Effect of PEG grafting density on non-covalent interactions

As described, increasing PEG grafting density increases non-covalent interactions until a plateau is reached, as shown in Fig. 5(a) and (b). To understand this better, we can replot these data as the fraction of PEG repeat units interacting with the PAMAM dendrimer as a function of total PEG chains. More specifically, the dependent variable is the fraction of PEG chain oxygen atoms hydrogen bonding with PAMAM primary amines, as all PEG chains are methoxy-capped leading to an extra oxygen-containing ether group per chain. To represent this, a ratio is calculated between the total PEG oxygen of all covalently bound mPEG chains and the total number of non-covalent interactions, as determined using eqn (4). Assuming the non-covalent interactions are hydrogen bonds, a value of one indicates that all PEG oxygen are forming hydrogen bonds with PAMAM, whereas a value of zero indicates that none of the PEG oxygen are forming hydrogen bonds with PAMAM.

**Fig. 6:**
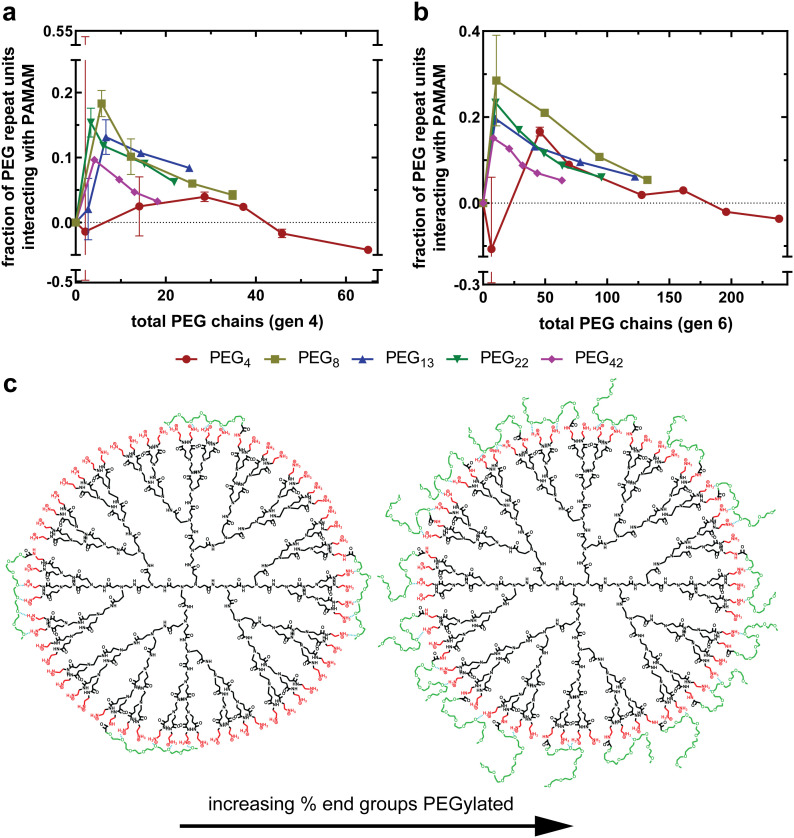
Increasing PEG grafting density decreases the fraction of PEG repeat units able to form non-covalent interactions with PAMAM amines. The fraction of all PEG repeat units non-covalently interacting with the underlying PAMAM surface for both generation 4 PAMAM (a) and generation 6 PAMAM (b) as a function of the percent of end groups PEGylated. For all generation-PEG length combinations, the fraction of PEG repeat units interacting decreases as a PEG density increase. (c) A visual representation of how increasing the number of PEG chains grafted onto the surface significantly impacts the steric hindrance between PEG chains.

Fig. 6(a) and (b) compares the fraction of all PEG oxygen atoms non-covalently interacting with PAMAM primary amines as a function of percent end groups PEGylated for generation four and six formulations, respectively. For all generation-chain length combinations, a maximum in PEG–PAMAM interactions is at low PEG grafting densities (<15%). As the PEG grafting density increases beyond this maximum, the fraction of PEG oxygen interacting with PAMAM primary amines decreases substantially.

At low grafting densities, PEG chains are able to interact freely with the PAMAM surface (Fig. 6(c), left). These interactions allow PEG chains to associate closely with the surface, facilitating thermodynamically favorable non-covalent interactions between PAMAM and PEG. As the grafting density increases, neighboring PEG chains, now in closer proximity, sterically repel one another (Fig. 6(c), right). The result is a force balance between attraction to PAMAM and repulsion from neighboring PEG. As the grafting density increases, the steric repulsion force increases while the electrostatic attraction remains constant, resulting in fewer interactions between PEG oxygen and PAMAM primary amines. This relationship between PEG–PAMAM interactions and PEG grafting density has been obtained in computational work, where it was observed as an increase in conjugate radius of gyration as PEG chains disassociate with the surface and extend into solution.^24^

The maximum in fraction of PEG oxygen interacting with primary amines is at a larger grafting density for shorter PEG chains than longer PEG chains (Fig. 6(a) and (b)). This further suggests that PEG chains are sterically interacting at grafting densities beyond the maximum. Longer PEG chains will begin interacting at a lower PEG grafting density, as their statistical radii are larger than that of shorter PEG chains. This results in the maximum observed interactions occurring at a lower PEG grafting density.

## Conclusions

In summary, this study developed a fluorescence-based method of determining the relative electrostatic binding affinity of PEGylated, cationic PAMAM dendrimers to an anionic matrix. By comparing these data to acetylated PAMAM formulations, the non-covalent interactions between covalently bound PEG chains and the underlying PAMAM surface were experimentally probed. Results on a library of PEG–PAMAM conjugates demonstrated that increasing PEG grafting density decreases the electrostatic binding affinity between cationic dendrimer and anionic matrix. This decrease in binding affinity is a result of reduced primary amines through chemical modification and shielding of charged, primary amines through non-covalent interactions with PEG. Increasing PEG chain length further reduces electrostatic binding affinity by increasing the non-covalent shielding of charged amines. When probed further, it was found that all PEG chain lengths tested have the largest number of non-covalent interactions with the PAMAM surface per chain at low grafting densities. As grafting densities increase, steric hindrance between neighboring PEG chains prevent individual PEG chains from interacting strongly with the surface. These findings coincide closely with computational findings in previous studies.^24^ This work is focused on the interactions between the grafted chains on the dendritic nanoparticle and the underlying dendrimer surface, in contrast to work on particle–particle interactions involving grafted chains. It is possible that these intraparticle findings might also impact how one considers interparticle interactions between grafted nanoparticles, as properties such as particle adhesion and composite toughness have been shown to be dependent on the conformation of grafted polymers in complex ways.^49–51^

While this study focused on PEGylated dendrimers and articular cartilage, we believe the protocol contained here can be adapted to other cationic carrier-anionic matrix systems, as long as certain criteria are met. For the system presented here, questions remain about the impact these findings have on the drug delivery properties of dendritic nanocarriers through anionic matrices. Preliminary findings suggest transport through anionic matrices is enhanced by the non-covalent interactions between PEG and PAMAM, but, ultimately, an extensive study probing various drug delivery properties must be conducted in order to truly understand the impact of these PEG–PAMAM interactions on the utility of PEGylated dendrimers as a drug delivery system. The information from our conclusions gives the field insight into how PEG influences the surface of cationic dendrimers, allowing for a greater understanding of how and why PEG affects various delivery properties.

## Conflicts of interest

There are no conflicts to declare.

## Supplementary Material

SM-019-D2SM01698B-s001
