# Supplementary material for: Charge shielding effects of PEG bound to NH2-terminated PAMAM dendrimers – an experimental approach
Source: Soft Matter. 2023 Mar 23;19(16):3033–46. doi: 10.1039/d2sm01698b (PMC10131161; doi:10.1039/d2sm01698b)
Supplement: SM-019-D2SM01698B-s001 [file SM-019-D2SM01698B-s001.pdf]

Electronic Supplementary Material (ESI) for Soft Matter.
This journal is © The Royal Society of Chemistry 2023

Charge shielding effects of PEG bound to NH_2-terminated PAMAM dendrimers – an experimental approach

Brandon M. Johnston[1,2], Alan J. Grodzinsky[3,4,5], Paula T. Hammond[1,2*]

[1]Department of Chemical Engineering, Massachusetts Institute of Technology, 77 Massachusetts Ave, Cambridge MA, 02139

[2]Koch Institute for Integrative Cancer Research, 500 Main St, Cambridge MA, 02142

[3]Department of Biological Engineering, Massachusetts Institute of Technology, 77 Massachusetts Ave, Cambridge MA, 02139.

[4]Department of Electrical Engineering and Computer Science, Massachusetts Institute of Technology, 77 Massachusetts Ave, Cambridge MA, 02139.

[5]Department of Mechanical Engineering, Massachusetts Institute of Technology, 77 Massachusetts Ave, Cambridge MA, 02139.

*To whom correspondence should be addressed: hammond@mit.edu

Supplementary Information

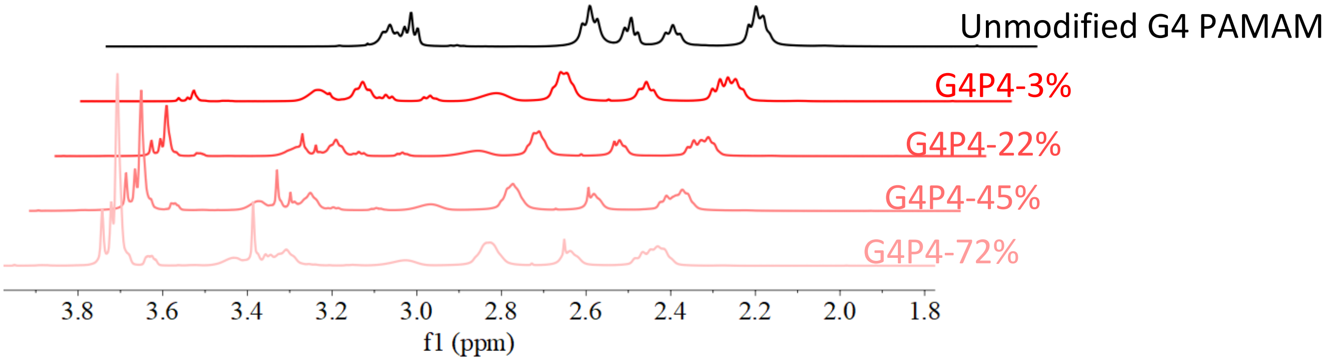

Fig. S1. Representative ^{1}H-NMR spectra for a library of PEGylated generation 4 PAMAM dendrimers. The PEG grafting density is calculated by comparing the characteristic PAMAM methylene peak at 2.4-2.5 ppm to the characteristic ethylene peak of PEG at 3.7-3.8 ppm.

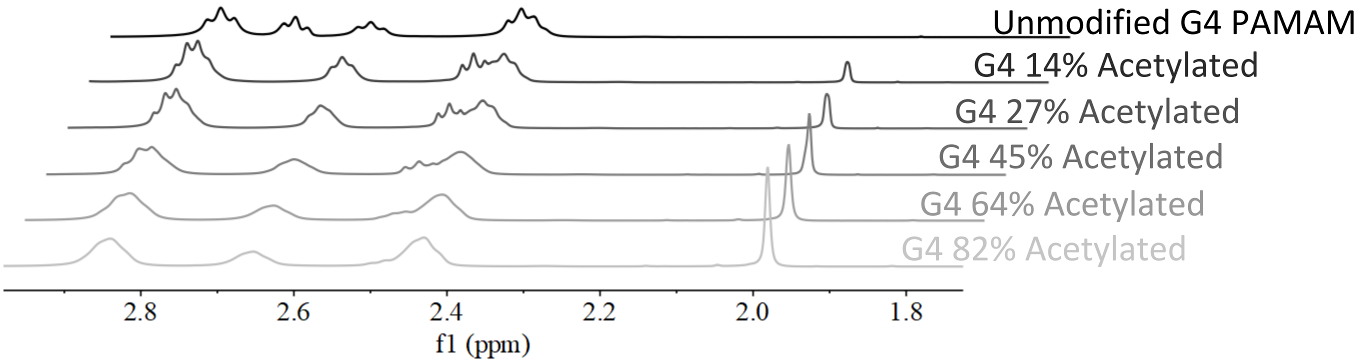

Fig. S2. Representative ^{1}H-NMR spectra for a library of acetylated generation 4 PAMAM dendrimers. The degree of acetylation is calculated by comparing the characteristic PAMAM methylene peak at 2.4-2.5 ppm to the characteristic methyl peak of the acetyl group at 2.0 ppm.

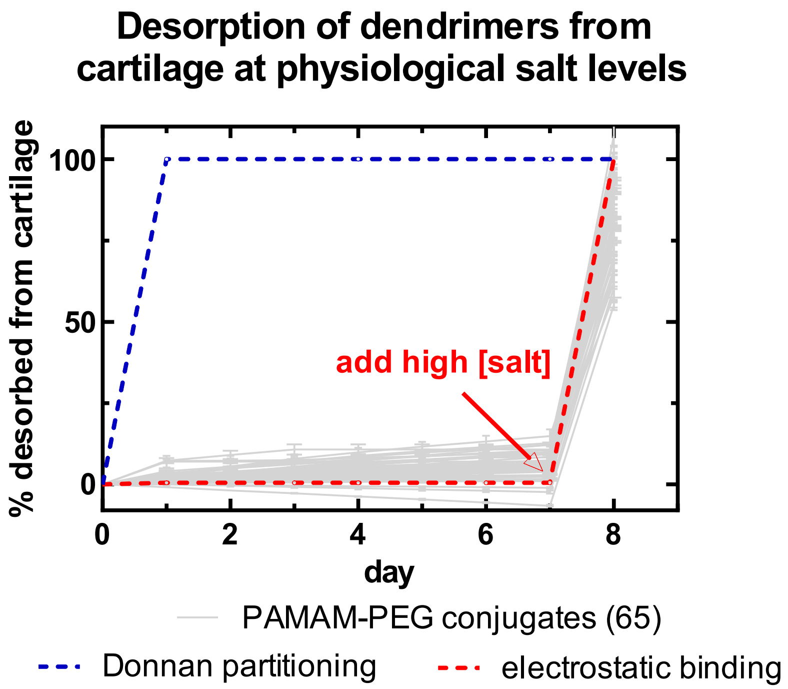

Fig. S3. Kinetics of desorption from *ex vivo* bovine cartilage for all conjugates tested. After binding of PEG-PAMAM conjugates to *ex vivo* cartilage, explants were incubated in 1x PBS for seven days, and the 1x PBS solution was replaced and fluorescence was measured every day. No formulation significantly desorbed from cartilage until a high salt concentration (10x PBS) was introduced.

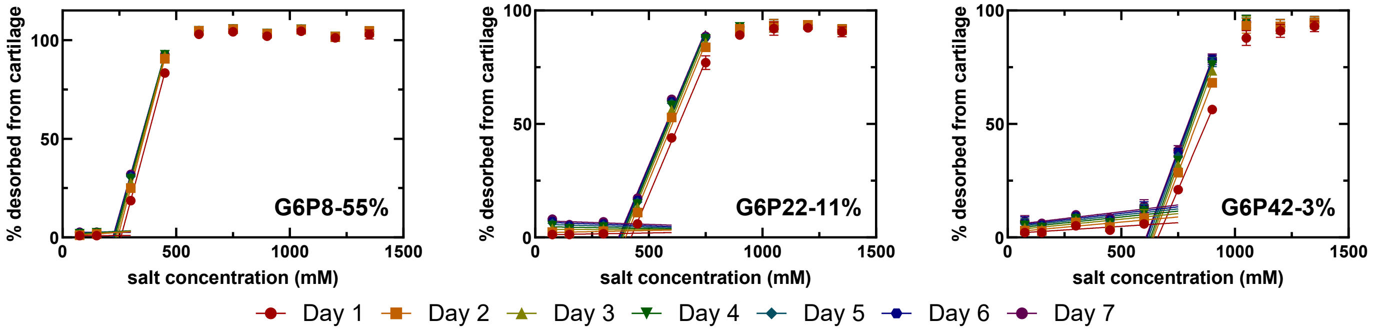

Fig. S4. Stability of *ex vivo* bovine cartilage explants and kinetics of desorption at high salt concentrations. Over a seven day period of removing and replacing the salt solutions of the critical salt concentration experiment, the results remained unchanged. This supports that kinetic effects of desorption can be ignored and the results are purely thermodynamic. Additionally, this supports that the cartilage explants remain intact and able to electrostatically bind with dendrimers for the entire seven day period.

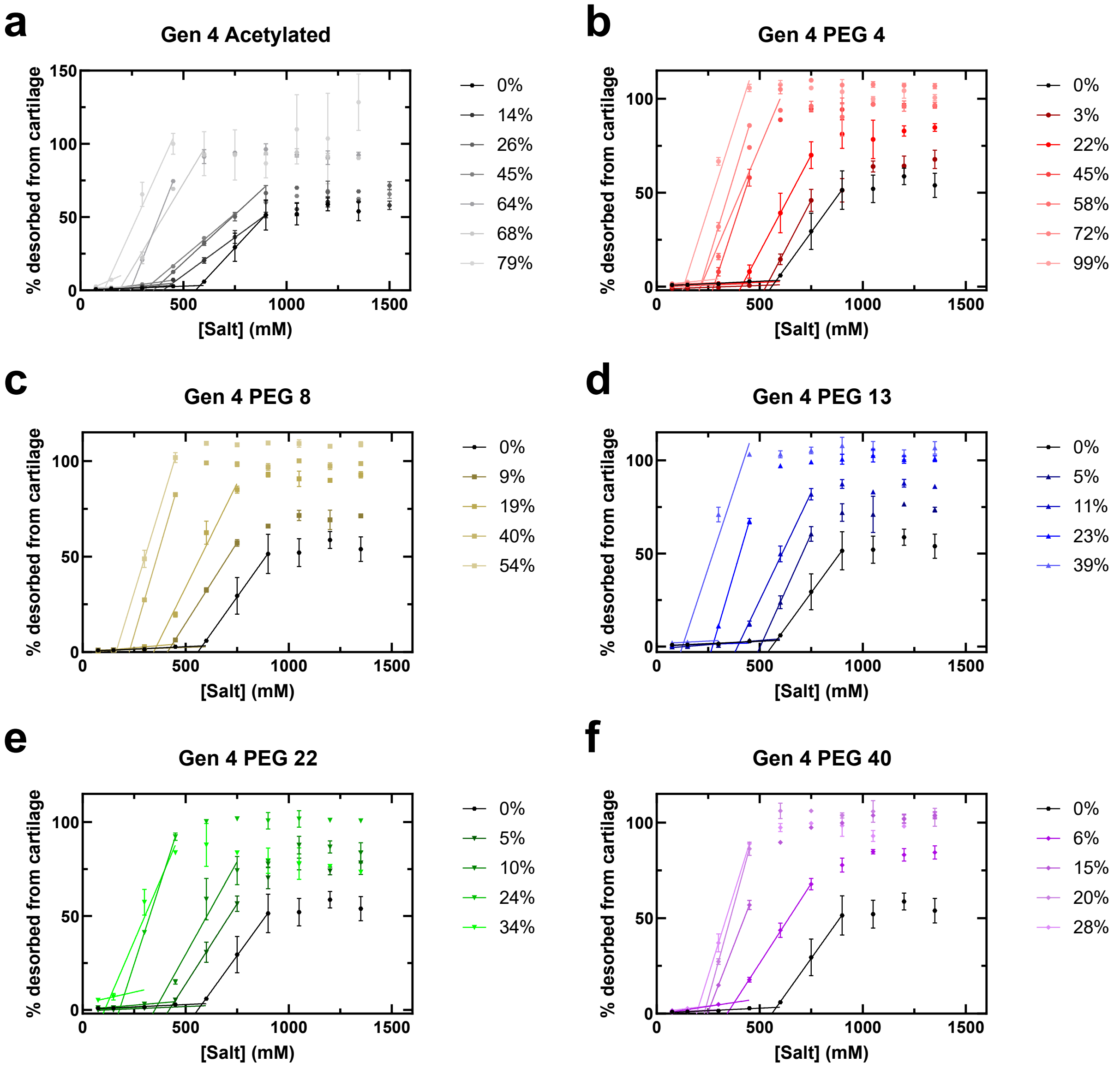

Fig. S4. Raw data from PEG screening assay for generation 4 formulations probing the desorption from cartilage explants at different salt concentrations. Critical salt concentration is determined by the onset of increasing desorption of dendrimer from cartilage, or where the lines of best fit shown here intersect. The order is as follows: (a) acetylated Gen 4, (b) Gen 4 PEG 4, (c) Gen 4 PEG 8, (d) Gen 4 PEG 13, (e) Gen 4 PEG 22, and (f) Gen 4 PEG 40.

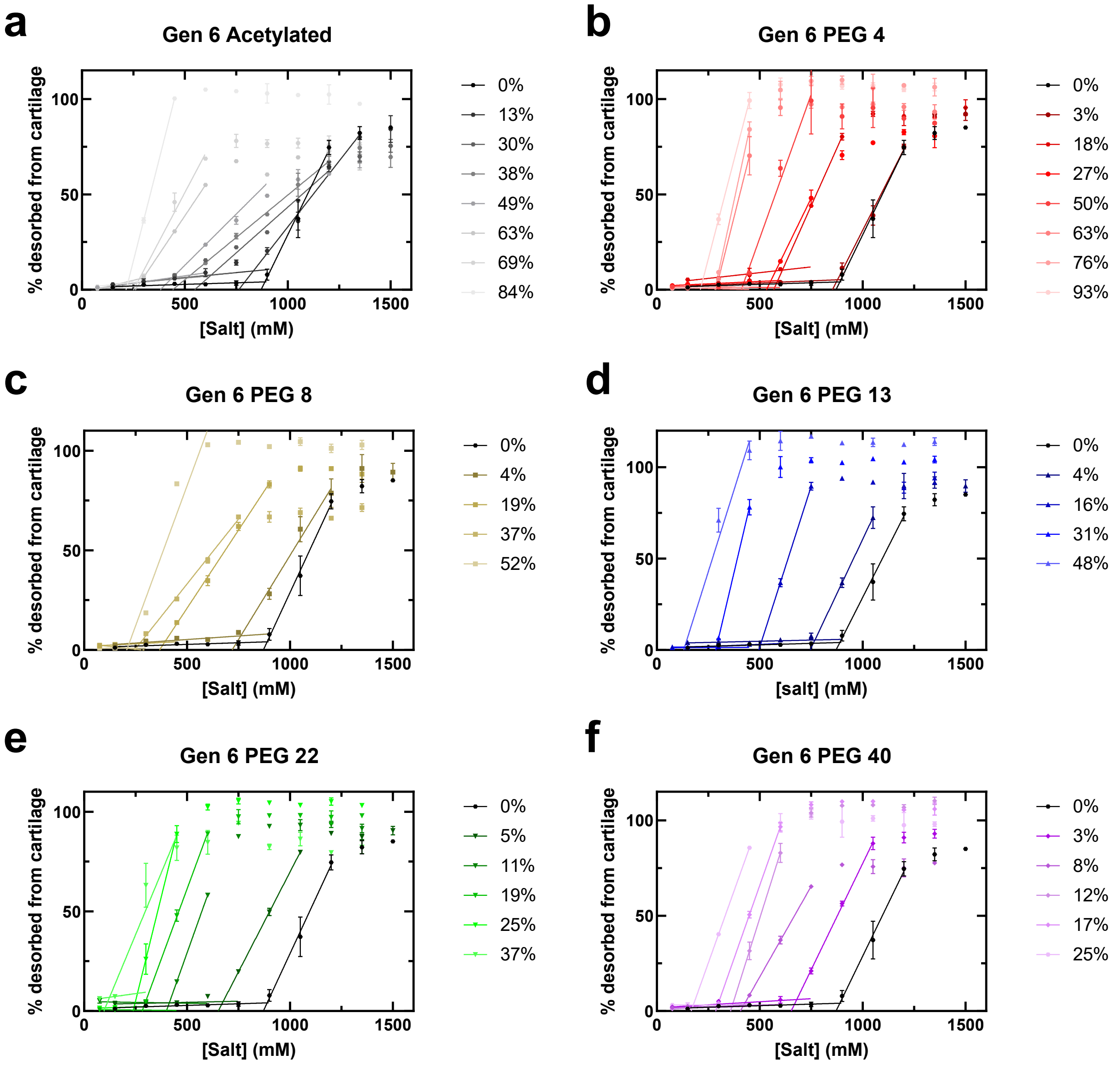

Fig. S5. Raw data from PEG screening assay for generation 6 formulations probing the desorption from cartilage explants at different salt concentrations. Critical salt concentration is determined by the onset of increasing desorption of dendrimer from cartilage, or where the lines of best fit shown here intersect. The order is as follows: (a) acetylated Gen 6, (b) Gen 6 PEG 4, (c) Gen 6 PEG 8, (d) Gen 6 PEG 13, (e) Gen 6 PEG 22, and (f) Gen 6 PEG 40.

Table S1. Pertinent values for application of Alexander-de Gennes theory to PEG-PAMAM conjugates

Generation	n_{amines}	*a*, dendrimer radius (nm)[a]	$4\pi a^2$, surface area (nm^2)	*x*, PEG degree of polymerization	*N*, PEG Kuhn segments[b]	$\langle r^2 \rangle^{1/2}$, PEG Flory radius (nm)[c]	$\Sigma_{overlap}$	$PEG_{overlap}$ #	$PEG_{overlap}$ %
Gen 4	64	2.092	55.0	4	1.9	1.11	0.82	**45**	**70.1**
				8	3.7	1.68	0.36	**20**	**30.5**
				13	6.1	2.25	0.20	**11**	**17.0**
				22	10.3	3.08	0.11	**6**	**9.1**
				42	19.7	4.54	0.05	**3**	**4.2**
Gen 6	256	3.193	128.1	4	1.9	1.11	0.82	**105**	**40.8**
				8	3.7	1.68	0.36	**46**	**17.8**
				13	6.1	2.25	0.20	**25**	**9.9**
				22	10.3	3.08	0.11	**14**	**5.3**
				42	19.7	4.54	0.05	**6**	**2.4**

[a]Porcar, *J Phys Chem B*, 2008
[b]Fully extended repeat unit length = 0.3556 nm
[c]PEG Kuhn length = 0.76

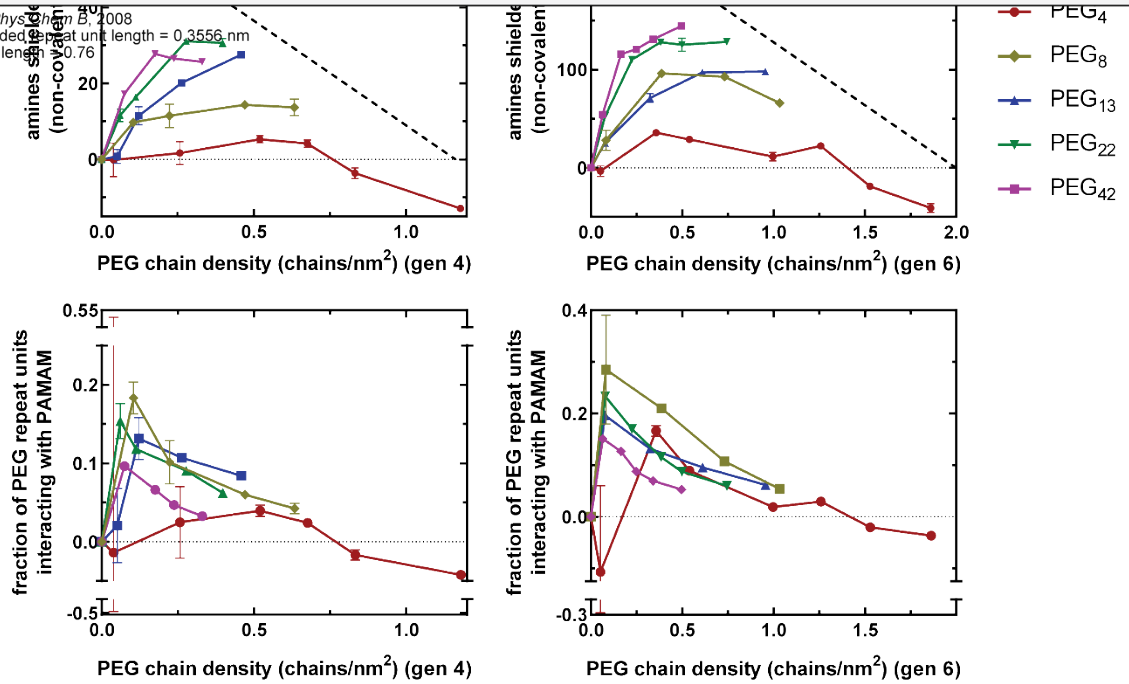

Fig. S6. Data recapitulated as a function of PEG chain density expressed as chains per nm². Accessible charged amines on the underlying PAMAM surface as a function of the PEG chain density (chains/nm²) for generation 4 (a) and generation 6 (b) PAMAM dendrimers. Vertical lines represent the PEG overlap chain density for the color corresponding PEG chain length. Amines shielded through non-covalent interactions between PEG and PAMAM as a function of PEG chain density for generation 4 PAMAM (c) and generation 6 PAMAM (d). The black, dotted line represents the maximum number of non-covalently shielded amines possible for a given PEG chain density. The fraction of all PEG repeat units non-covalently interacting with the underlying PAMAM surface for both generation 4 PAMAM (a) and generation 6 PAMAM (b). For all generation-PEG length combinations, the fraction of PEG repeat units interacting decreases as a PEG density increase. Each point represents the mean and standard deviation of 2 biologic replicates with n=1 technical replicate per animal.
